# Stroke risk study based on deep learning-based magnetic resonance imaging carotid plaque automatic segmentation algorithm

**DOI:** 10.3389/fcvm.2023.1101765

**Published:** 2023-02-24

**Authors:** Ya-Fang Chen, Zhen-Jie Chen, You-Yu Lin, Zhi-Qiang Lin, Chun-Nuan Chen, Mei-Li Yang, Jin-Yin Zhang, Yuan-zhe Li, Yi Wang, Yin-Hui Huang

**Affiliations:** ^1^Department of Neurology, The Second Affiliated Hospital of Fujian Medical University, Quanzhou, Fujian, China; ^2^Department of Neurology, Anxi County Hospital, Quanzhou, Fujian, China; ^3^Department of Neurology, Jinjiang Municipal Hospital, Shanghai Sixth People’s Hospital Fujian Campus, Quanzhou, Fujian, China; ^4^Department of CT/MRI, The Second Affiliated Hospital of Fujian Medical University, Quanzhou, China

**Keywords:** stroke risk, MRI carotid plaque, deep learning, transfer learning, YOLO V3

## Abstract

**Introduction:**

The primary factor for cardiovascular disease and upcoming cardiovascular events is atherosclerosis. Recently, carotid plaque texture, as observed on ultrasonography, is varied and difficult to classify with the human eye due to substantial inter-observer variability. High-resolution magnetic resonance (MR) plaque imaging offers naturally superior soft tissue contrasts to computed tomography (CT) and ultrasonography, and combining different contrast weightings may provide more useful information. Radiation freeness and operator independence are two additional benefits of M RI. However, other than preliminary research on MR texture analysis of basilar artery plaque, there is currently no information addressing MR radiomics on the carotid plaque.

**Methods:**

For the automatic segmentation of MRI scans to detect carotid plaque for stroke risk assessment, there is a need for a computer-aided autonomous framework to classify MRI scans automatically. We used to detect carotid plaque from MRI scans for stroke risk assessment pre-trained models, fine-tuned them, and adjusted hyperparameters according to our problem.

**Results:**

Our trained YOLO V3 model achieved 94.81% accuracy, RCNN achieved 92.53% accuracy, and MobileNet achieved 90.23% in identifying carotid plaque from MRI scans for stroke risk assessment. Our approach will prevent incorrect diagnoses brought on by poor image quality and personal experience.

**Conclusion:**

The evaluations in this work have demonstrated that this methodology produces acceptable results for classifying magnetic resonance imaging (MRI) data.

## 1. Introduction

Global mortality and morbidity are primarily caused by cardiovascular disease (CVD), and 17.9 million fatalities each year globally are attributable to CVD, or 31% of all deaths (1). The primary factor for CVD and upcoming cardiovascular events is atherosclerosis. Atherosclerosis development and plaque formation in the vasculature, including the coronary and carotid arteries, are the primary causes of CVD (2). Plaque rupture or ulceration frequently leads to the development of a thrombus, which may embolize or occlude the lumen, blocking blood flow and resulting in myocardial infarction or stroke (3). The plaque is seen and screened using a variety of medical imaging techniques, the most popular of which are magnetic resonance imaging (MRI), computed tomography (CT), and ultrasound (US).

Recently, because of significant inter-observer variability, the texture of carotid plaques, as seen on ultrasonography, is variable and challenging to classify with the human eye. In order to determine the mechanical qualities caused by the influence of the lipid core and calcification within a plaque, numerical simulation is also employed to define the distribution and components of the plaque structure (4). Compared to CT and ultrasonography, high-resolution MR plaque imaging provides naturally superior soft tissue contrasts, and a combination of various contrast weightings may yield more insightful data. Two further advantages of MRI include operator independence and the absence of radiation. However, other than preliminary research on MR texture analysis of basilar artery plaque, there is currently no information addressing MR radiomics on carotid plaque (5).

Since medical images contain a plethora of information, many automatic segmentation and registration approaches have been investigated and proposed for use in clinical settings. Deep learning technology has lately been used in various industries to evaluate medical images, and it is particularly good at tasks like segmentation and registration. Several CNN architectures have been suggested that feed whole images with increased image resolution (6). Fully CNN (fCNN) was developed for segmenting images and was first introduced by Long et al. (7). However, fCNNs produce segmentations with lower resolution than the input images. That was brought about by the later deployment of convolutional and pooling layers, both of which reduce the dimensionality. For multiple sclerosis lesion segmentation, Brosch et al. (8) suggested using a 3-layer convolutional encoder network to anticipate segmentation of the same resolution as the input pictures. Kamnitsas et al. (9) used a deep learning technique to categorize ischemic strokes. Roy and Bandyopadhyay (10) examined Adaptive Network-based Fuzzy Inference System (ANFIS), a suggested method for categorizing cancers into five groups. The Gray-Level Co-Occurrence Matrix (GLCM) was used to obtain characteristics that were used to categorize and segment tumors using pre-trained AlexNet (11). In this research, we used various pre-trained deep learning models to the automatic segmentation of MRI carotid plaque for Stroke risk assessment. Deep learning networks have recently been repeatedly suggested for enhancing segmentation performance in medical imaging. Segmentation performance can be improved by combining segmentation and classification, regression, or registration tasks (12).

## 2. Proposed methodology

**Figure 1:**
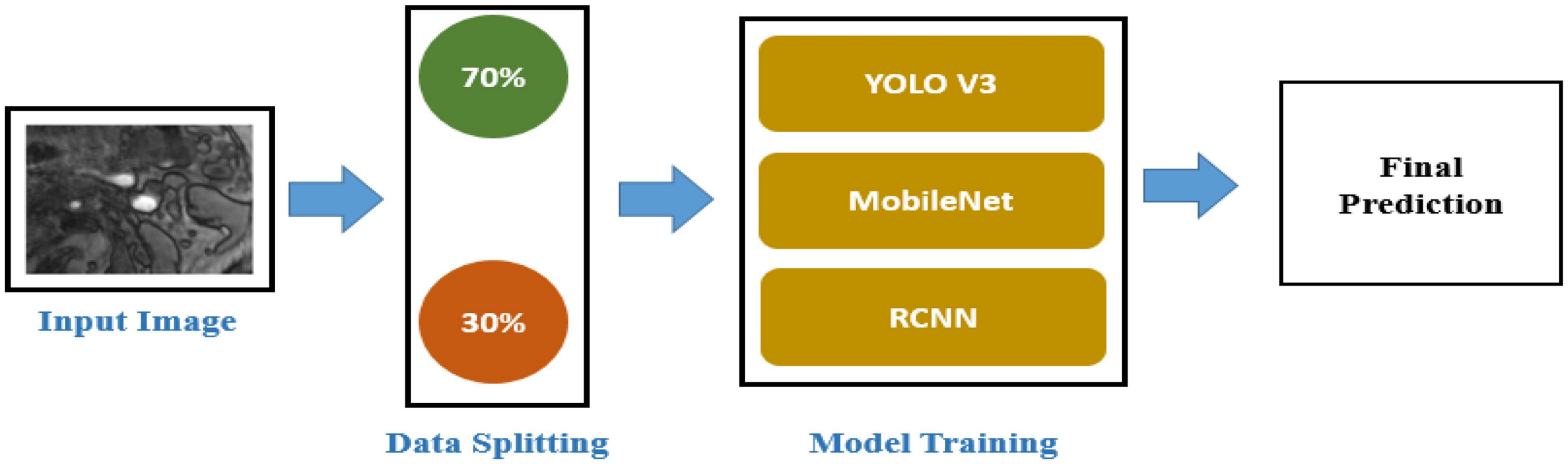
Proposed framework.

For automatic segmentation of MRI scans to detect carotid plaque for stroke risk assessment, there is a need for a computer-aided autonomous framework to classify MRI scans automatically. Deep learning technology has recently permeated several areas of medical study and has taken center stage in modern science and technology (13). Deep learning technology can fully utilize vast amounts of data, automatically learn the features in the data, accurately and rapidly support clinicians in diagnosis, and increase medical efficiency (14). In this research, we proposed a deep learning framework based on transfer learning to detect carotid plaque from MRI scans for stroke risk assessment. We used YOLO V3, Mobile Net, and RCNN pre-trained models, fine-tuned them and adjusted hyperparameters according to our dataset. All experiments in this paper are conducted on Intel(R) Celeron(R) CPU N3150 @ 1.60 GHz. The operating system is Windows 64-bit, Python 3.6.6, TensorFlow deep Learning framework 1.8.0, and CUDA 10.1. The proposed framework to address the mentioned research problem is shown in Figure 1.

### 2.1. Data collection and statistics

The data of 265 patients were collected from the Second Affiliated Hospital of Fujian Medical University, in which 116 patients have a high risk of plaques, and the remaining 149 patients have a stable condition and have a low chance of plaques. The detailed process and parameters for the data collection are described in the following subsections.

#### 2.1.1. Inclusion criteria

Carotid artery stenosis detected by ultrasound, CTA, MRA, and other numerical simulations (15) methods needs to be identified; ultrasound and CTA indicate plaque formation on the wall, regardless of whether the patient has clinical symptoms; carotid artery is not found by other imaging examinations Significant stenosis, but clinical symptoms: TIA and cerebral infarction of unknown cause. Magnetic resonance carotid artery scans were performed.

#### 2.1.2. Scanning parameters

Philips 3.0 T MRI with 8-channel phased array surface coil dedicated for carotid artery assessment. Instruct the patient to lie down, keep calm during the scanning process, avoid swallowing, and place the jaw and neck in the center of the 8-channel phased array surface coil. First, the bilateral carotid arteries were scanned by coronal thin slice T2WI scanning, and the images were reconstructed to obtain the shape and stenosis position of the carotid arteries. The sequence and imaging parameters are as follows:

- Rapid gradient echo (3D MERGE): 3D motion sensitized driven equilibrium prepared; fast gradient echo (turbo field echo); duration of repetition (TR)/time of echo (TE) 9/4.2 ms; field of view (FOV) 25,164 cm^3^; spatial resolution (SR) 0.80.80.8 mm^3^; flip angle 6°; imaging time 4 min.
- 3D simultaneous noncontract angiography and intraplaque hemorrhage (3D SNAP): TFE TR/TE 10/4.8 ms, FOV 25 × 16 × 4 cm^3^, spatial resolution 0.8 × 0.8× 0.8 mm^3^, flip angle 11°/5°, imaging time 5 min.
- 3D time of flight (TOF): fast field echo (FFE) TR/TE 20/4.9 ms, FOV 16 × 16 × 4 cm^3^, spatial resolution 0.6 × 0.6 × 2 mm^3^, flip angle 20°, imaging time 6 min. Axial 3DTOF, fast spin echo (FSE)-based T1WI and T2WI scans were performed in the longitudinal range of 20–24 mm (10–12 layers) with the stenosis as the center, supplemented by fat suppression (FS). The positions of the patients’ T1WI, T2WI, and 3DTOF sequences were kept consistent, and the images of patients with carotid plaques were selected for further study. The images were post-processed by the MRI-VPD system, and Plaque View software was used to analyze the properties and components of carotid plaques. All analysis and measurement steps were performed independently by three senior radiologists. The above examinations were obtained with the consent of the patients and their families and signed informed consent.

**Figure 2:**
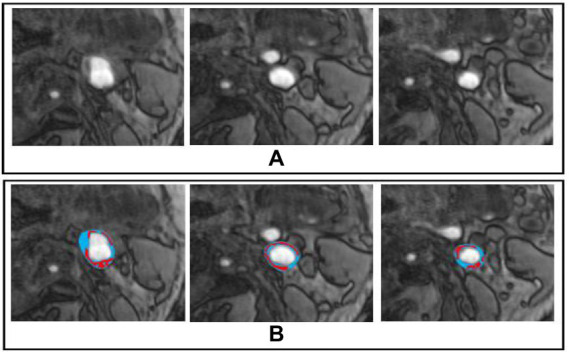
Sample Dataset, whereby **(A,B)** represents the original and marked image for carotid plaque, respectively.

The sample dataset is shown in Figure 2. Furthermore, we also show the dataset statistics in the table for better understanding as shown in Table 1.

**Table 1:** Detailed statistics of the dataset.

Total patients	High risk of plaques	Low risk of plaques
265	116	149

### 2.2. Yolo V3

A deep learning network called YOLO identifies and categorizes objects in the input photos. The object detection task entails locating each object on the input image and classifying it according to the bounding box that surrounds it (16). A single Convolutional Neural Networks (CNNs) architectural model is used in the YOLO deep learning network to concurrently localize the bounding boxes of objects and classify their class labels from all images. The YOLO loss for each box prediction comprises coordinate loss due to the box prediction not covering an object as described in Eq. 1. Where o_i_ is the output value, and t_i_ is the target value.


(1) $$BC\;Eloss=-\frac{1}{n}\times \underset{i}{\sum }\left(t_{i}\times \log\left(o_{i}\right)+\left(1-t_{i}\right)\times \log\left(1-o_{i}\right)\right)$$

The primary addition here is that YOLO V3 is able to extract more valuable semantic data from the up-sampled features during training. Table 2 displays the whole model architecture and hyper parameter details.

**Table 2:** YOLO V3 hyper parameters.

Type	Filters	Size	Output
Convolutional	32	3 × 3	256 × 256
Convolutional	64	3 × 3/2	128 × 128
Convolutional	32	1 × 1	
Convolutional	64	3 × 3	
Residual			128 × 128
Convolutional	128	3 × 3/2	64 × 64
Convolutional	64	1 × 1	
Convolutional	128	3 × 3	
Convolutional	256	3 × 3/2	32 × 32
Convolutional	128	1 × 1	
Convolutional	256	3 × 3	
Residual			32 × 32
Convolutional	512	3 × 3/2	16 × 16
Convolutional	256	1 × 1	
Convolutional	512	3 × 3	
Residual			16 × 16
Convolutional	1,024	3 × 3/2	8 × 8
Convolutional	512	1 × 1	
Convolutional	1,024	3 × 3	
AVG pool		GLOBAL	
Connected		1,000	
Soft max			

### 2.3. MobileNet

The MobileNet model is the first mobile computer vision model for TensorFlow and is designed for mobile applications, as its name suggests. MobileNet uses depth-wise separable convolutions and features filters/kernels that are $D_{k}\times D_{k}\times 1$ . It significantly lowers the number of parameters when compared to a network with conventional convolutions of the same depth in the nets, and the convolution operation is represented in Eq. 2


(2) $$\mathrm{Total}\;\mathrm{no}\;\mathrm{of multiplications}=M\;x\;D_{k}{}^{2}\;x\;D_{p}{}^{2}$$

The result of this is lightweight deep neural networks. The new architecture requires fewer operations and parameters to accomplish the same filtering and combination procedure as a typical convolution. The entire model architecture and hyperparameter details are displayed in Table 3, where each line represents a sequence of one or more identical layers (modulo stride) repeated n times and an expansion factor of *t*. Both layers share the output channel number c for the identical sequence. Every sequence starts with a stride, and all subsequent layers also employ a stride. All spatial convolutions employ 3×3 kernels.

**Table 3:** Mobile net model hyper parameters.

Input	Type	t	c	n	s
2,242 × 3	conv2d	-	32	1	2
1,122 × 32	Bottleneck	1	16	1	1
1,122 × 16	Bottleneck	6	24	2	2
562 × 24	Bottleneck	6	32	3	2
282 × 32	Bottleneck	6	64	4	2
142 × 64	Bottleneck	6	96	3	1
142 × 96	Bottleneck	6	160	3	2
72 × 160	Bottleneck	6	320	1	1
72 × 320	conv2d 1 × 1	–	1,280	1	1
72 × 1,280	avgpool 7 × 7	–	–	1	–
1 × 1 × 1,280	conv2d 1 × 1	–	k	–	

### 2.4. R-CNN

The sliding-window paradigm is the foundation of the previous localization strategy for CNN, however, it struggles to achieve acceptable localization precision when working with more convolutional layers. The, the authors suggested using the region paradigm to address the CNN localization issue (17). Three modules make up the R-CNN design principle (1). The first module aims to produce a set of category-independent region recommendations using selective search (18), a search method that combines the best aspects of exhaustive search and segmentation intuitions.

One of the best techniques for reducing overfit is increasing the training dataset’s size. The training images were automatically resized using an augmented image dataset. Our pre-trained deep learning model avoids over-fitting by using the dropout layer.

## 3. Results and discussion

Global mortality and morbidity are primarily caused by cardiovascular disease (CVD), and 17.9 million fatalities each year globally are attributable to CVD, or 31% of all deaths. Atherosclerosis is the primary factor for CVD and upcoming cardiovascular events (19). The main causes of CVD are atherosclerosis development and plaque production in the vasculature, including the coronary and carotid arteries. Since medical images contain a plethora of information, many automatic segmentation and registration approaches have been investigated and proposed for use in clinical settings. Recently, deep learning technology has been used in various industries to analyze medical images. In this research, we proposed a deep learning framework based on transfer learning to detect MRI scans into a carotid plaque for stroke risk assessment. We used YOLO, Mobile Net, and RCNN pre-trained models, fine-tuned them and adjusted hyperparameters according to our problem. The data of 265 patients were collected from the Second Affiliated Hospital of Fujian Medical University, in which 116 patients have a high risk of plaques, and the remaining 149 patients have a stable condition and have a low risk of plaques. Then, using a random selection approach, we divide the data in the ratio of 70% for training and 30% for the testing set.

**Figure 3:**
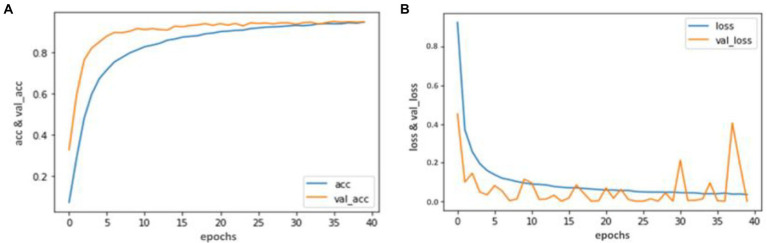
Accuracy and Loss graph using the YOLO V3. **(A)** Representing the training and Validation accuracy while **(B)** representing the training and validation loss of YOLO V3 model according to our dataset.

**Figure 4:**
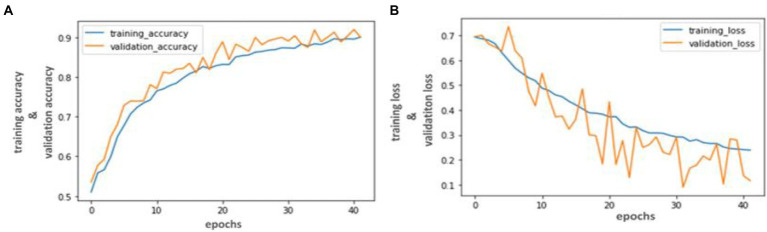
Accuracy and Loss graph using the RCNN. **(A)** representing the training and validation accuracy while **(B)** representing the training and validation loss of RCNN model according to our dataset.

**Figure 5:**
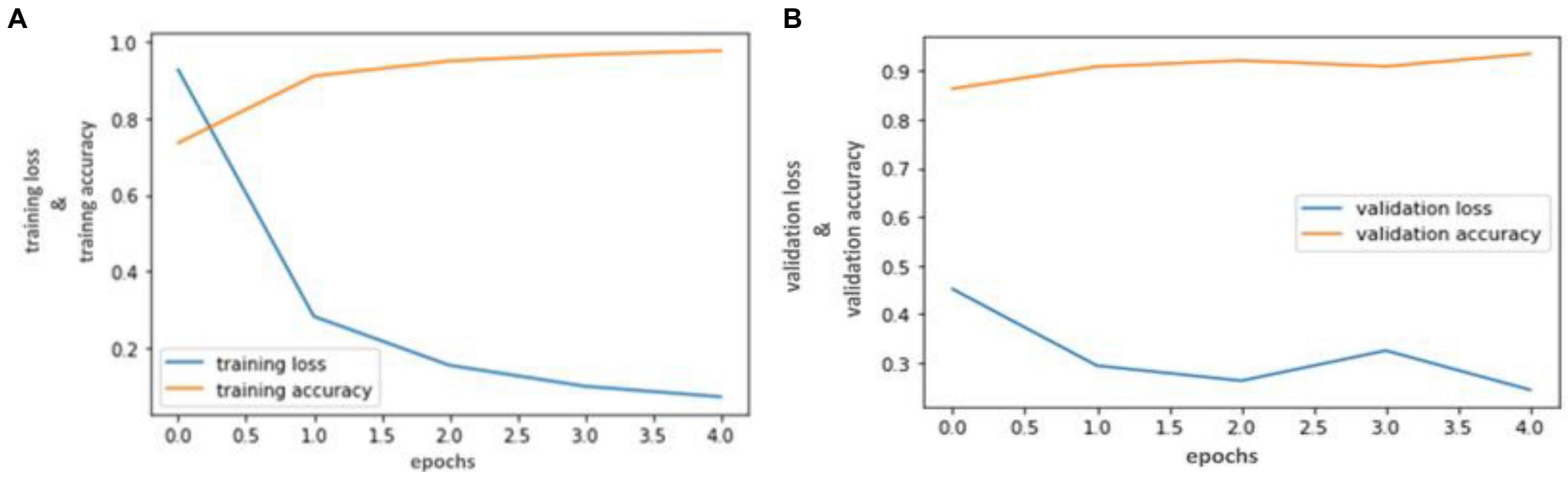
Accuracy and Loss graph using the Mobile Net. **(A)** Representing the training loss and training accuracy while **(B)** representing the validation loss and validation accuracy of the Mobile Net model according to our dataset.

Our trained YOLO model achieved 94.81% accuracy, RCNN achieved 92.53% accuracy, and Mobile Net achieved 90.23% in identifying carotid plaque from MRI scans for stroke risk assessment. We used accuracy and loss graphs to evaluate the performance of our model. According to our dataset, Figures 3, 4A,B respectively, show the training and validation accuracy and training and validation loss for the YOLO V3 Mobile Net models. Similar to Figure 5, which uses the RCNN model to identify carotid plaque from MRI scans for stroke risk assessment, Figure 5A shows the training loss and training accuracy, and Figure 5B shows the validation loss and validation accuracy.

**Figure 6:**
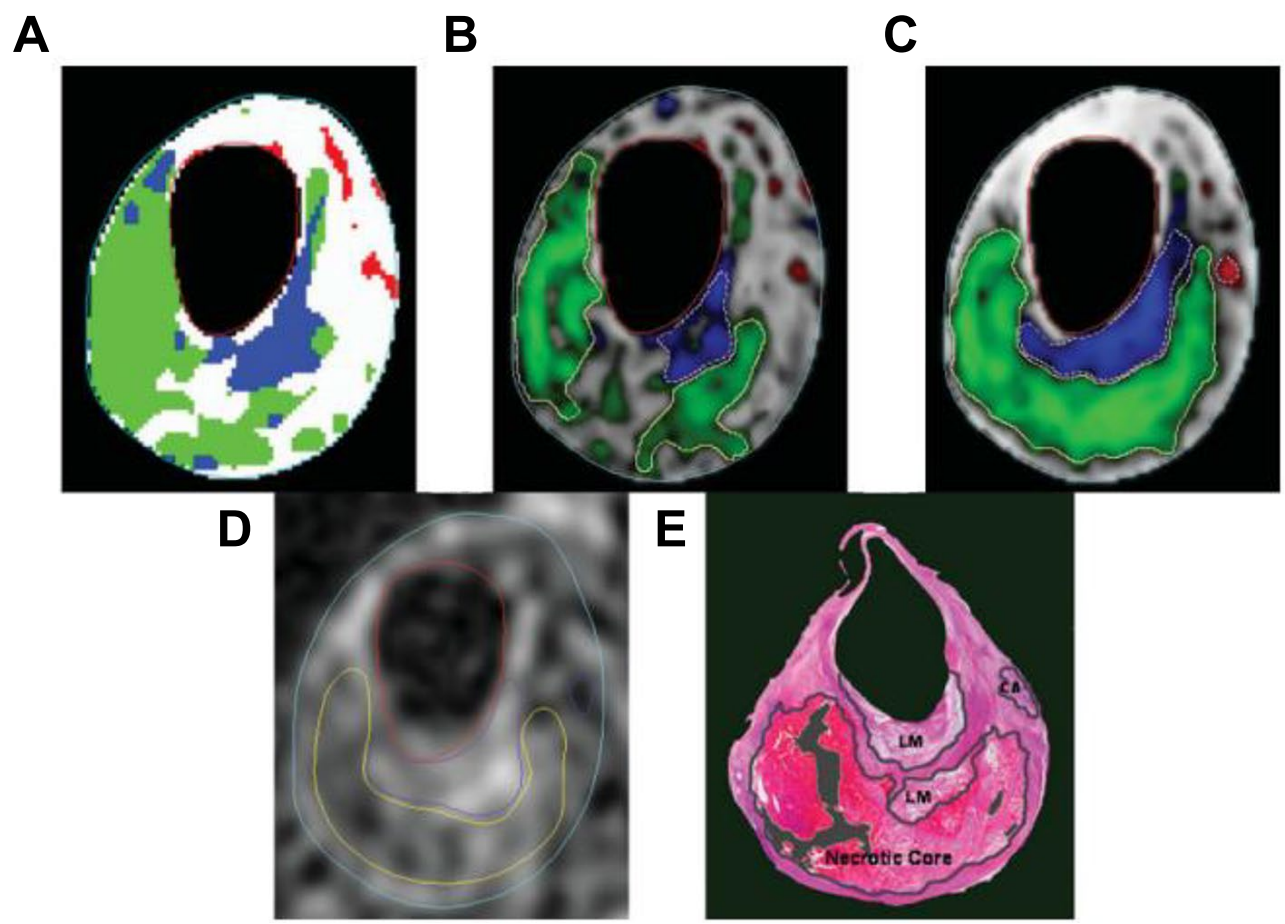
The following segmentation results are displayed on a T2-weighted image. **(A)** Automatic labeling result by Gaussian classifier. **(B)** Probability map and region contours based only on intensity, with a necrotic core in green, calcification in a red, loose matrix in blue, and fibrous tissue in gray. **(C)** Corresponding results with morphologic information. **(D)** Manual segmentation result. **(E)** Corresponding histology specimen used to direct contour placement in **(D)** dark areas.

Table 4 lists the classification accuracies in terms of sensitivity and specificity for each pixel in the testing set. Both blinded manual and automated segmentation yield similar results, showing high specificities for all tissue categories and great sensitivity for fibrous tissue. In contrast to the loose matrix, which has very poor sensitivity, necrotic core and calcifications sensitivity is good. This metric is pessimistic for small locations, like the majority of calcifications and confusion matrix, which can mainly cause a slightly lower sensitivity. The segmentation result in Figure 6 serves as an illustration of this observation.

**Table 4:** Pixel-wise segmentation accuracy.

	Sensitivity	Specificity
Necrotic	0.75	0.92
Calcification	0.65	0.98
loose matrix	0.51	0.97
Fibrous tissues	0.88	0.78

**Figure 7:**
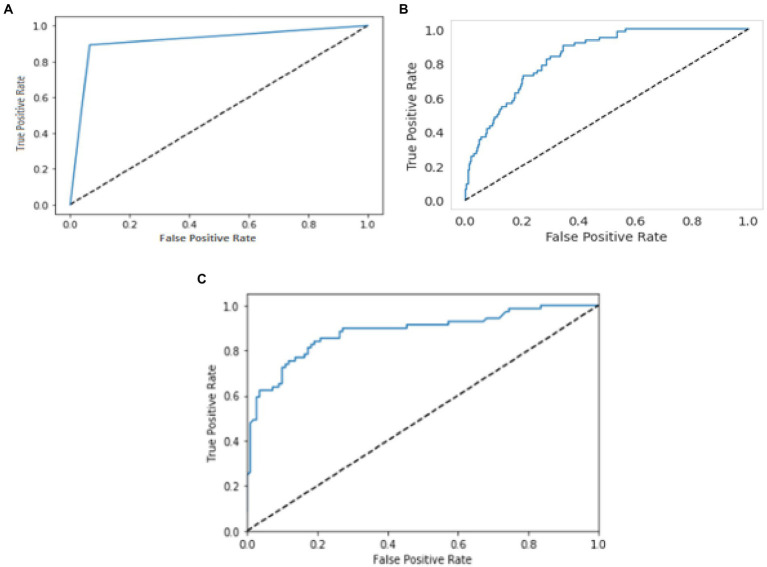
ROC curves for three models. Whereby **(A–C)** represents the performance of the YOLO V3, MobileNet, and RCNN, respectively, to detect carotid plaque from MRI scans for stroke risk assessment.

The relationship or trade-off between clinical sensitivity and specificity for each potential cut-off for a test or set of tests is usually depicted graphically using ROC curves. The performance of two or more diagnostic tests is compared using the ROC curve (20), which is used to evaluate a test’s overall diagnostic performance. It is also used to choose the best cut-off value for assessing whether a disease is present. Figure 7 represents the performance of three models by using the ROC curve. Here (a) illustrates the performance of the YOLO V3 model to detect carotid plaque from MRI scans for stroke risk assessment. Similarly, (b) represents the performance of Mobile Net in terms of the confusion matrix, and (c) illustrates the performance of the RCNN model to detect carotid plaque from MRI scans for stroke risk assessment.

In this research, we proposed a deep learning framework based on transfer learning to detect carotid plaque from MRI scans for stroke risk assessment. We used to detect carotid plaque from MRI scans for stroke risk assessment pre-trained models, fine-tuned them, and adjusted hyperparameters according to our problem. The proposed framework assists the radiologist in early and accurate carotid plaque detection from MRI scans for stroke risk assessment. Our proposed framework also improves the diagnosis and addresses other challenges in MRI diagnosis due to various issues. Furthermore, we have compared our proposed framework performance with the previously proposed approach shown in Table 5 (23–26).

**Table 5:** Comparative accuracy of the proposed approach with previous proposed studies.

Reference study	Approach	Accuracy (%)
Jamthikar et al. (21)	Machine learning models	93
Qian et al. (22)	DL models	90.6
Our proposed framework	Pre-trained models	**94.81**

Highlight the accuracy of our model.

## 4. Conclusion

In this study, we concluded that deep learning-based methods for stroke risk assessment are the most promising and successful. Our trained YOLO model achieved 94.81% accuracy, RCNN achieved 92.53% accuracy, and Mobile Net achieved 90.23% in identifying carotid plaque from MRI scans for stroke risk assessment. Using accuracy, loss graphs, and ROC curves, we evaluated the performance of our model and found that the suggested framework performed better. Our approach will prevent incorrect diagnoses brought on by poor image quality and personal experience. The evaluations in this work have demonstrated that this methodology produces acceptable results for classifying MRI data. Future applications may employ extreme learning as a more sophisticated classifier for plaque categorization issues.

## 5. Limitations and future work

Deep learning requires a large amount of data to improve performance and avoid over-fitting. It is difficult to acquire medical imaging data of low-incidence serious diseases in general practice. Due to differences in patients and the appearance of the prostate, future work will focus on testing the model with a more extensive data set. The, even though the results of studies have the potential for deep learning associated with different kinds of images, additional studies may need to be carried out clearly and transparently, with database accessibility and reproducibility, in order to develop valuable tools that aid health professionals.

## Data availability statement

The original contributions presented in the study are included in the article/supplementary material, further inquiries can be directed to the corresponding author/s.

## Ethics statement

The studies involving human participants were reviewed and approved by Second Affiliated Hospital of Fujian Medical University. The patients/participants provided their written informed consent to participate in this study. Written informed consent was obtained from the individual(s) for the publication of any potentially identifiable images or data included in this article.

## Author contributions

Y-HH, Z-JC, and Z-QL: study design. Y-YL and M-LY: data interpretation. Y-HH and Y-ZL: manuscript production. YW, J-YZ, and Y-FC take responsibility for the integrity of the data analysis. All authors contributed to the article and approved the submitted version.

## Funding

This study was funded by Quanzhou City Science and Technology Program of China (No. 2020N003).

## Conflict of interest

The authors declare that the research was conducted without any commercial or financial relationships construed as a potential conflict of interest.

## Publisher’s note

All claims expressed in this article are solely those of the authors and do not necessarily represent those of their affiliated organizations, or those of the publisher, the editors and the reviewers. Any product that may be evaluated in this article, or claim that may be made by its manufacturer, is not guaranteed or endorsed by the publisher.
